# Emergence of spatially structured populations by area‐concentrated search

**DOI:** 10.1002/ece3.9528

**Published:** 2022-11-30

**Authors:** Thotsapol Chaianunporn, Thomas Hovestadt

**Affiliations:** ^1^ Department of Environmental Science, Faculty of Science Khon Kaen University Khon Kaen Thailand; ^2^ Biocenter, Department of Animal Ecology and Tropical Biology, Theoretical Evolutionary Ecology Group University of Würzburg Würzburg Germany

**Keywords:** area‐concentrated search, individual‐based model, metapopulation, spatially structured population

## Abstract

The idea that populations are spatially structured has become a very powerful concept in ecology, raising interest in many research areas. However, despite dispersal being a core component of the concept, it typically does not consider the movement behavior underlying any dispersal. Using individual‐based simulations in continuous space, we explored the emergence of a spatially structured population in landscapes with spatially heterogeneous resource distribution and with organisms following simple area‐concentrated search (ACS); individuals do not, however, perceive or respond to any habitat attributes per se but only to their foraging success. We investigated the effects of different resource clustering pattern in landscapes (single large cluster vs. many small clusters) and different resource density on the spatial structure of populations and movement between resource clusters of individuals. As results, we found that foraging success increased with increasing resource density and decreasing number of resource clusters. In a wide parameter space, the system exhibited attributes of a spatially structured populations with individuals concentrated in areas of high resource density, searching within areas of resources, and “dispersing” in straight line between resource patches. “Emigration” was more likely from patches that were small or of low quality (low resource density), but we observed an interaction effect between these two parameters. With the ACS implemented, individuals tended to move deeper into a resource cluster in scenarios with moderate resource density than in scenarios with high resource density. “Looping” from patches was more likely if patches were large and of high quality. Our simulations demonstrate that spatial structure in populations may emerge if critical resources are heterogeneously distributed and if individuals follow simple movement rules (such as ACS). Neither the perception of habitat nor an explicit decision to emigrate from a patch on the side of acting individuals is necessary for the emergence of such spatial structure.

## Graphical abstract

We investigate the emergence of a spatially structured population in landscapes with spatially heterogeneous resource distribution and with organisms following simple area‐concentrated search (ACS) by using individual‐based simulations in continuous space. We found that neither the perception of habitat nor an explicit decision to emigrate from a patch on the side of acting individuals are necessary for the emergence of spatial structure.

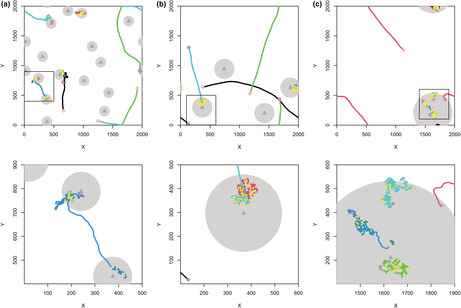

## 1 INTRODUCTION

The idea of spatially structured population, namely metapopulation, patchy population, mainland–island system, or source–sink systems, has become a very powerful concept in ecology, raising interest in research areas like dispersal ecology (Hanski, 2012; Lambin et al., 2012; With, 2004) or population genetics (Haig, 1998; Harrison & Hastings, 1996; Hastings & Harrison, 1994; Manel et al., 2003; Montgelard et al., 2014). The concept also had a strong impact on the development of conservation concepts (Akçakaya et al., 2007; Hanski & Simberloff, 1997; Olivieri et al., 2016; Thomas, 1995). However, these concepts may be more a “construct” of human observers with their tendency to categorize observations—yet not necessarily reflect the biology underlying the emergence of spatial population structure. In particular, there is no guarantee that the organisms under investigation have a perception (or a “concept”) of habitat patches or that they at any time “decide” to emigrate from a habitat patch and disperse. Current approaches typically assume the concept to be valid but do not necessarily explain its emergence from first principle.

Another issue with the metapopulation and other spatially structured population concepts is that they do not explicitly account for movement behavior and dispersal that emerges from it (Bowler & Benton, 2005; Hanski, 1999; Hawkes, 2009) even though dispersal is arguably the most important ingredient of the concepts. In particular, it is not guaranteed that dispersal occurs (only) because of the particular “decision” to disperse, eventually at a certain moment in the life cycle. Dispersal, i.e., the movement of individuals between habitat patches, may also come about by routine movement, e.g., during foraging.

Over the last decades, research has progressed in better understanding what drives the movement of individuals searching for critical resources (Bartoń & Hovestadt, 2013; Hawkes, 2009; Pyke, 2015). Indeed, a rich literature exists of investigating and understanding rules of foraging movement at the individual and local level (Benhamou, 2007; Hills et al., 2013; James et al., 2011; Plank & James, 2008; Pyke, 2015; Viswanathan et al., 1999). In fact, searching for some critical commodity like food, mating partners, or nest sites may be the motivation underlying the far majority of any movement in mobile animals. Some studies (e.g., Getz & Saltz, 2008; Nathan et al., 2008) thus proposed a conceptual framework for movement ecology that considers the interplay among mechanistic components of movement: the internal state, motion, navigation capacities of the individual, and the external factors affecting movement. The underlying idea of this and other concepts is the proposition that individuals usually have a cause or motivation to move and that they collect and process information to steer their movement; an approach that questions the wide‐held assumption in metapopulation models that movement and consequently dispersal would be random. Some authors have already created movement models with some or all of those components of movement (e.g., Avgar et al., 2013; Barton et al., 2009; Bartoń & Hovestadt, 2013; Bartumeus & Catalan, 2009; Benhamou, 1992; Fagan et al., 2013, 2017; Fronhofer et al., 2013; Fryxell et al., 2008; McNamara et al., 2006; Olsson & Brown, 2010; Reynolds, 2012; Van Moorter et al., 2009). However, because such models have mostly been used to understand how movement rules affect individual foraging success, we are still only beginning to understand how rules for routine movement might scale up to patterns at the population and landscape levels, i.e., to the level of spatially structured populations.

In this article, we propose that features of a spatially structured population and possibly of a metapopulation can emerge if animals follow simple movement rule like simple area‐concentrated search (ACS; also named “area‐restricted search”) and if critical (and searched) resources are themselves heterogeneously distributed. Area‐concentrated search, a type of “state‐dependent correlated random walk,” has previously been used in many ecological studies (such as Bartoń & Hovestadt, 2013; Benhamou, 1992, 2004; Kareiva & Odell, 1987; as the “Mushroom Hunt Model” in Railsback & Grimm, 2012; Turchin, 1991). According to the ACS, a change to searching behavior as indicated by low directionality (correlation) of movement (and low movement speed) might be affected by, e.g., (perceived) habitat attributes per se as in Turchin (1991), diffuse cues like odor or smell of prey (e.g., Nolting et al., 2015), or an individual's internal state (e.g., hunger level, previous foraging success, or recent encounters with prey). Any of these movement rules, as well as others with more sophisticated modes of context‐dependent movement, might have similar effects, however.

Area‐concentrated search movement strategies may approach the efficiency of an unconstrained optimal forager (Adler & Kotar, 1999) and seem to occur in many different species (reviewed in Dorfman et al., 2022), like mallards (at very small spatial scale; Klaassen et al., 2006), wandering albatrosses that respond to habitat cues per se (Weimerskirch et al., 2007), amoeba (Van Haastert & Bosgraaf, 2009), where straight movement is triggered by starvation, or ladybird beetles that respond to prey encounters (Nakamuta, 1985).

Here, we simulate the ACS movement of foraging organisms in a landscape with differently clustered resource distribution (single large cluster vs. many small clusters and different resource density) and explore how this influences the distribution of individuals in space, foraging success, and the movement between resource clusters (viz. habitat patches). We speculate that a spatially heterogeneous resource distribution and such a simple movement rule are sufficient to generate the different attributes of a spatially structured population or metapopulation: namely (i) spatially clustered distribution of individuals in areas of high resource concentration, (ii) different movement pattern inside and outside patches—searching behavior within, but straight‐line movement outside of habitat patches, (iii) emigration rate depending on patch quality—reduced emigration from large or high‐quality habitat patches vs. elevated emigration from small‐ or poor‐quality patches. Some authors have explored such movement models previously (Bartoń & Hovestadt, 2013; Benhamou, 1992, 2004; Nolting et al., 2015; Turchin, 1991) but were interested in specifying how such rules affect foraging success or movement attributes in different sections of a landscape and not on the emerging spatial distribution of individuals at the population level which is the focus of this study.

## 2 MATERIAL AND METHODS

We implement a simple model simulating the movement of resource‐searching individuals (ACS) in a continuous landscape with heterogeneous resource distribution; both the position of individuals and resources are thus continuous point coordinates. We investigate how resource distribution affects the spatial distribution (density) of individuals and the movement (dispersal) of individuals between resource clusters. Our simulation ignores birth and death events, but the model implicitly accounts for the diffuse effect of competition over resources on foraging behavior.

### 2.1 Spatial distribution of resources and scenarios

We simulated foraging movement in a square landscape of area 4 × 10^6^ (2000 × 2000) squared spatial units with resources distributed within it. In the simulations, we created *k* resource clusters within the landscape as continuous spatial point pattern with points generated by the Matérn Cluster Point Process, using R version 3.5.2, library spatstat version 1.58–2 (Baddeley & Turner, 2005). Clusters were generated with daughter points (resources) distributed according to a random uniform distribution on a disk around parent points with *g* as radius of the clusters and *u* as resource density per area unit. Thus, $\bar{R}=g_{i}^{2}\pi \times u$ was the expected number of resource items per cluster, and the expected number of resources items in the landscape was $k\times \bar{R}$. The center of each parent point was distanced at least 3 *g* units apart from the center of any other parent point to avoid cluster overlapping. The landscapes were wrapped into a torus in both dimensions to avoid edge effects and mimic a landscape of infinite dimension. Across scenarios, the number of resource clusters was increased from *k* = 1 to *k* = 16 clusters, whereas the radius of clusters (*g*) was reduced from 320 (at *k* = 1) to 80 (at *k* = 16) so that the total area covered by resource clusters was identical in all scenarios (*c*. 8% of total area). The average resource density in resource clusters was varied from *u* = 0.01 to *u* = 1.27 resources per unit area (see Table 1 for more details). A summary of all model and simulation parameters and their values can be found in Table 1.

**TABLE 1:** Definition and ranges of parameters values used.

Symbol	Description	Values
*k*	Number of resource clusters within the landscape	16, 8, 4, 2, 1
*g*	Radius of clusters corresponding to *k*	80, 80√2, 160, 160√2, 320
*u*	Resource density	0.01, 0.02, 0.04, 0.08, 0.16, 0.32, 0.64, 1.27 resources items per unit area
*d*_min_	Minimum value for correlation of turning angles of consecutive steps	0.01
*d*_max_	Maximum value for correlation of turning angles of consecutive steps	1 (corresponds to straight‐line movement)
*f*	Shape parameter	3
*h*	Half‐saturation constant	200 (0 for SLM and 10,000 for CRW)
*p*	Step length of movement	1
*c*	Perception radius	1

### 2.2 Movement rule

The movement of each individual was modeled as an ACS. Here, we implemented the simplest of such possible rules, assuming that individual *i* moved straighter, the longer the time interval in which it did not find a food item was, i.e., the longer the searching time ${∆}_{S,i}$ was (reviewed in Bartoń & Hovestadt, 2013; see Benhamou, 1992); generally, such models have been shown to be efficient foraging strategies (Benhamou, 1992; Pyke, 2015) also in comparison to the much discussed Lévy walk (e.g., Nolting et al., 2015; Plank & James, 2008). Comparable movement was, for example, observed in starved amoeboid cells that move rather straight whereas well‐fed cells moved changed direction much more frequently (Van Haastert & Bosgraaf, 2009) but just as well in mammal species (Auger‐Méthé et al., 2016). At any moment *t*, and for any moving individual *i*, the turning angle between two consecutive steps was determined by drawing a random value from a wrapped circular normal distribution (Jammalamadaka & SenGupta, 2001) with mean 0 and standard deviation $d_{i,t}$(${∆}_{S,i}$) calculated as follows:

$$d_{i,t}\left(\Delta_{S,i}\right)=d_{\min}+\left(d_{\max}-d_{\min}\right)\cdot \left(1-\frac{\Delta_{S,i}^{\alpha }}{\left(\Delta_{S,i}^{\alpha }+h^{\alpha }\right)}\right).$$

**FIGURE 1:**
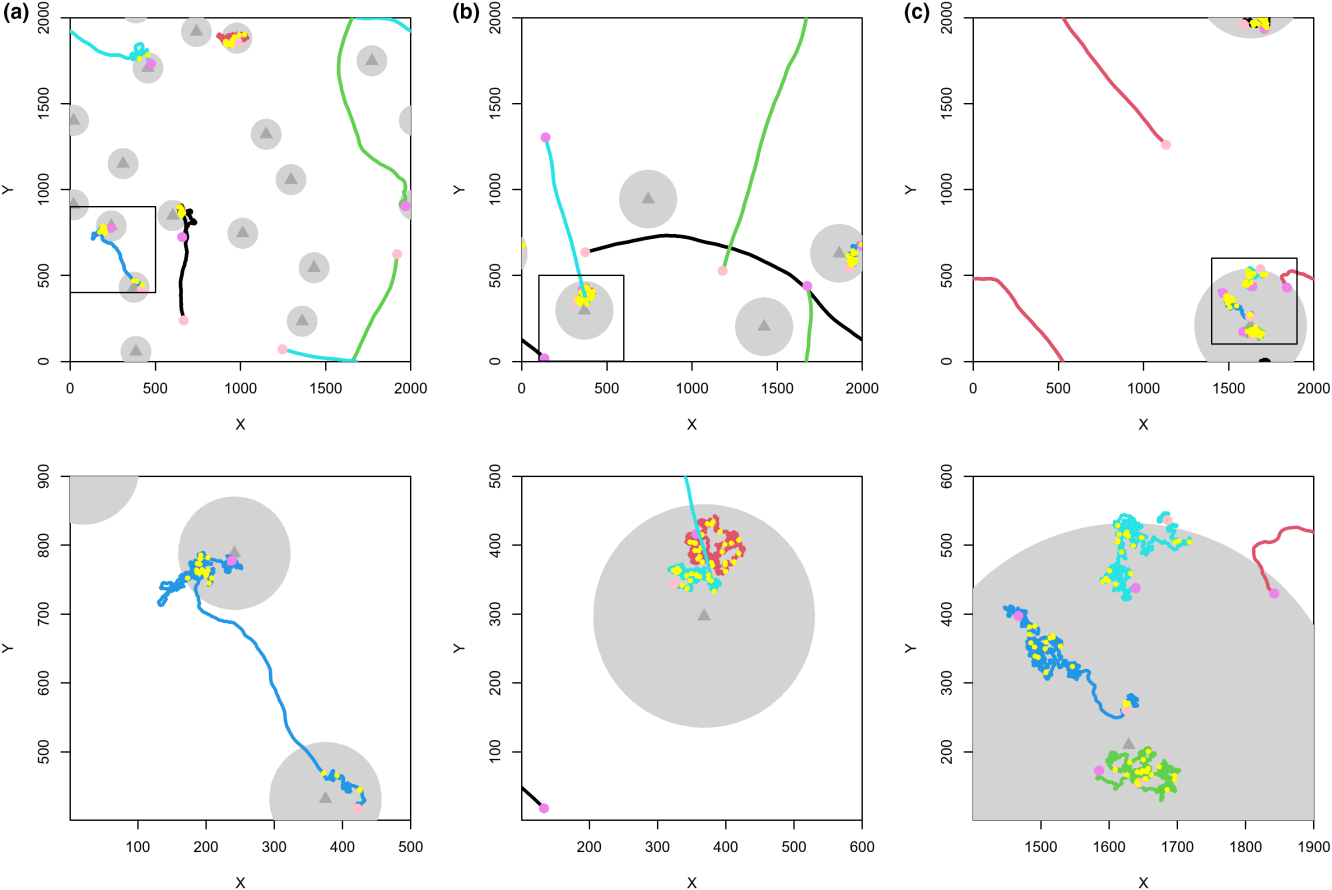
Examples of movement path of five individuals (five different color lines) in the landscape of different scenarios from last 2000 time steps. Pink points indicate the starting position of the movement, yellow points show the positions where resource items were harvested, and purple points are the end position of the movement. Large gray circles present the position and size of resource clusters, and the gray triangles show the positions of parent points. Upper figures present the movement paths in the whole landscape and lower figures indicate the movement paths in a section of the square area in the upper figures: (a) scenario with 16 clusters (*k* = 16) with size of 80 units (*g* = 180) and resource density of 0.16 (*u* = 0.16) resources per unit; (b) *k* = 4, *g* = 160, *u* = 0.16; (c) *k* = 1, *g* = 320, *u* = 0.16.

Consequently, $d_{i,t}$ ranges between $d_{\min}=0.01$ (nearly straight‐line movement) when $\Delta_{S,i}\gg h$ and $d_{\max}=1$ when $\Delta_{S,i}=0$, i.e., when the individual just found a food item. In the latter case, the movement became highly uncorrelated, and the individual performed area‐concentrated search. We used a wrapped circular normal distribution here because the normal distribution is common in nature. The parameter α is a shape parameter (in our simulations always α=3), and *h* is the half‐saturation constant (always h=200). The effects of parameter α and *h* on $d_{i,t}$ and on foraging success were described in Bartoń and Hovestadt (2013). In preliminary simulations, we tested different values of *h* (50≤h≤800) and α (1≤α≤20) and found that changes of either parameter value within these ranges did not qualitatively affect results. A value of α≈3 led, however, to maximum foraging success in the study by Bartoń and Hovestadt (2013). We thus kept these two‐parameter values constant in all main simulations. Examples of movement paths of individuals from simulations are shown in Figure 1.

Nonetheless, with *h* approaching extremes the nature of movement changes qualitatively. For this reason, we carried out—just for the four‐cluster scenario—further simulations with *h* = 0 (resulting in unconditional straight‐line movement; SLM) or *h* = 10,000, approximating an unconditional simple correlated random walk (CRW, see below).

### 2.3 Foraging

At each time step, each individual moved one step according to the movement rule described above. Individuals were moved in a random sequence to avoid priority benefits. The step length of movement (*p*) was constant and equal to 1 spatial unit. After movement, an individual immediately found all resource items within its perception radius (c=1 spatial unit, identical to the step length). All resource items within this radius were “foraged” and removed (the individual maintained its position, however). Following a movement step, the value of $\Delta_{S,i}$ for each individual was increased to $\Delta_{S,i}+1$ in case an individual did not find a resource item but was reset to $\Delta_{S,i}=0$ whenever the individual found a food item, thus initiating the ACS as described above.

After movement of all individuals, removed resource items were replaced by a same number of new items placed randomly as daughter points of randomly selected parent points according to the rules explained above (global replacement). With this global replacement, we implemented a global equilibrium assumption between resource production (regrowth) and consumption yet nonetheless allowing for the more short‐term depletion (competition) effects due to intense local harvesting.

### 2.4 Simulations and analysis

For each parameter combination (resource density and cluster size, see above), we carried out 10 replicates on 10 independently created landscapes. In each simulation, 80 individuals were released at random coordinates within resource clusters; in the added simulations with SLM and CRW, individuals were released at random positions within the landscape (for full comparison we also repeated the ACS simulations with this initialization, called ACS‐Random). The number of individuals simulated might affect some results in this study, in particular patch occupancy, but the main findings are not influenced as they are derived from individual attributes.

At initialization, $\Delta_{S,i}$ was set to $\Delta_{S,i}=500$ so that individuals started with nearly straight‐line movement. The initial direction of each individual was randomly selected from a uniform distribution between 0 and 2π. At each time step, individuals moved and foraged resource items as described above. All individuals were allowed to move for 10,000 steps, but all analyses described below are based on data collected over the last 2000 movement steps only.

At the beginning of each simulation, the expected number of resource items per cluster was equal to $\bar{R}$ (see above). Due to the global replacement of foraged resource items, the total number of resource items in the landscape was kept constant, and consequently, the average number of resource items per cluster remained at $\bar{R}$. However, the number of items in a single cluster could vary over time and degrade if the cluster was harvested intensively, i.e., by many individuals at the same time.

Effects of resource density and cluster size on the distribution of individuals and spatial structure of the system were evaluated in this study. For graphical presentation, the grand mean of 10 replicates are shown in figures with calculations based on the averages calculated across all individuals within single replicates. Foraging and movement behavior of individuals in different scenarios were compared according to (1) foraging success (=proportion of time steps when an individual harvests one or more resource items) and (2) total number of different clusters from which resource items were collected.

We defined immigration as a moment when an individual entered the area of a cluster (radius around a parent point) even without foraging success and emigration as the moment when an individual left away from this area. For analyzing the duration of movements within and between clusters, we noted the moments of emigration from and the moments of immigration into a patch. For (3) duration of visits to a patch (“patch visitation time”), we counted the time when an individual stayed within patch radius and for (4) duration of “patch searching time,” we counted the time when an individual was in the “matrix areas” between resource clusters. The data also allowed to calculate, however, the emigration events also contained short excursions away and back to a cluster similar to “foray loops” (a succession of progressively larger ellipsoidal loops) previously described in Conradt et al. (2003) and McIntire et al. (2013). We thus separated (5) excursions of less than 200 steps as “foray loops” from “long‐distance emigration events” in our analyses.

For determining the spatial structure of our system, we measured (6) patch occupancy (proportion of time patches contained at least one individual), (7) the percentage of individuals located in clusters, (8) the number of “successful” migration events, i.e., transitions from one cluster to another. For the analysis (3, 4, 5, 8), individuals that never entered a patch within the last 2000 time steps were excluded.

## 3 RESULTS

### 3.1 Foraging behavior and foraging success

In this study, we investigated the emergence of spatially structured population in the simulations with a simple movement rule of individuals in the system, the area‐concentrated search, in patch landscape with clustered resource distribution. We could observe features of spatially structured populations in our systems.

Examples of movement of individual in different scenarios are shown in Figure 1. In concordance with the principles underlying the ACS, two types of movement can be recognized in our simulations—searching for (or “dispersing between”) resource clusters and foraging within resource clusters. Straight‐line movement primarily (and obviously) occurred in the “matrix areas” between resource clusters, whereas foraging—characterized by more uncorrelated movement—occurred within resource clusters.

**FIGURE 2:**
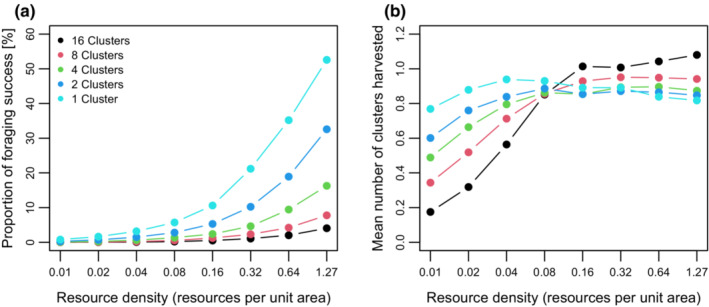
(a) Percentage of movement (time) steps with foraging success (harvesting one or more resource items) calculated over the last 2000 time steps and plotted over resource density. (b) Total number of clusters from which resource items were harvested during the last 2000 time steps plotted over resource density.

We represent foraging success of each individual by the proportion of time steps when an individual encountered resources (Figure 2a). Foraging success increased with increasing resource density (trivially) and decreasing number of resource clusters. Overall, individuals were more successful in a landscape with a single large resource cluster than in landscapes with many small clusters even though the total area covered by the clusters in different scenarios was equal. This effect was more pronounced at low resource density than at high resource density (e.g., at *u* = 0.01, the foraging success in the one‐cluster scenario was approximately 64‐fold higher than that in 16‐cluster scenario, while this difference was approximately 13‐fold at *u* = 1.27). When resource clusters were small and/or resource density was low, individuals often moved through clusters without encountering resource items within their perceptual range and thus maintaining their straight searching movement. In other words, individuals eventually did not “recognize” the presence of a resource aggregation if resource density was rather low, and clusters were small.

### 3.2 Patch visitation and patch searching

**FIGURE 3:**
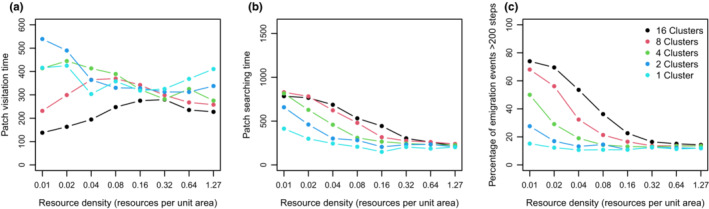
(a) Averaged duration of visits to a resource cluster (patch visitation time); (b) averaged duration of patch searches (patch searching time) during the last 2000 time steps plotted over resource density; (c) the percentage of emigration events that were longer than 200 steps (permanent emigration) plotted over resource density.

We generally expected that individuals would stay and forage longer for resources within a patch and also detected new patches easier when resources were dense than when resources were sparse, but our simulations provided more complex results. The mean number of clusters from which resources were collected was mostly <1 (this value included individuals that did not successfully reach any resource cluster) and smaller than the number of clusters they entered because some individuals did not detect resource item within clusters (Figure 2b). In the many small cluster scenarios (16 and 8 clusters), the number of clusters harvested continuously increased with increasing resource density (Figure 2b). The relationship between cluster size, resource density, and patch residence time (inverse of emigration rate) turned out to be complex. Generally, and expectedly, individuals resided longer in larger clusters, but as we observed for each cluster size different unimodal relationships with resource density, this ranking was not persistent across resource density (Figure 3a); the peak in the relationship shifts from higher to lower resource densities as patch size increases. For all cluster sizes, patch searching time declined with resource density, but the effect was more pronounced for small compared to large cluster scenarios (Figure 3b).

**FIGURE 4:**
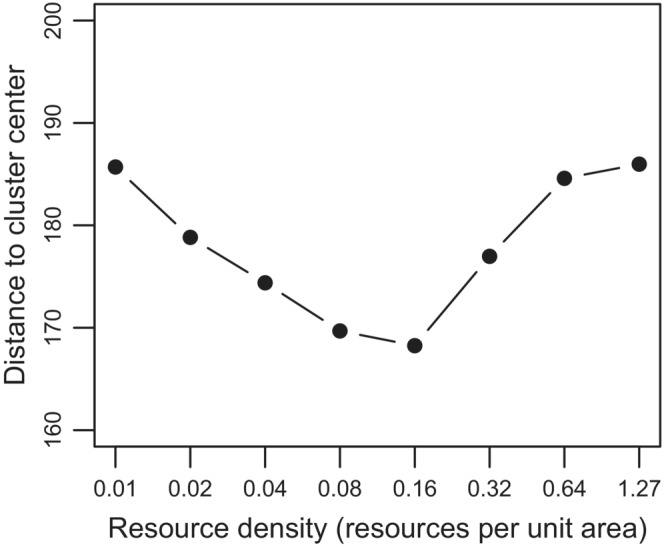
Averaged distance between the current location of an individual in cluster at the last time step and the parent point of the cluster (patch center) from the scenario with single cluster (patch radius = 320 spatial unit) plotted over resource density.

That residence times are shorter at low resource density is understandable as individuals will often keep the straight‐line movement as they might not encounter resources. However, to better understand the declines of residence time at high resource densities, we analyzed—just for the one‐cluster scenarios—the position of individuals within the resource cluster at the last time step. We found that the mean positions of individuals in scenarios with moderate resource density were closer to the center point (parent point) than those in scenarios with high and low resource density (Figure 4). In other words, individuals tended to penetrate deeper into a resource cluster (move closer to the patch center) with moderate resource density than in a cluster with high resource density because they were less likely to encounter a resource item near the edge of the cluster upon arrival than in high resource density scenarios. Consequently, the chance to move away from a cluster briefly after it was found was lower in the scenarios with intermediate resource density. In scenarios with high resource density, individuals foraged mainly close to the edge of a cluster with the associated risk of eventually leaving that cluster.

In addition, not all of these emigration events resulted in permanently leaving a resource cluster so that individuals eventually returned to the cluster they just left before, resulting in a “foray loop” (cf. Conradt et al., 2003). Using an arbitrary cutoff level of 200 time steps to separate between “permanent emigration” and foray loops, we recognize that with increasing resource density a larger proportion of emigration episodes falls into the foray loop category (Figure 3c). The results show that long‐distance emigration events occurred more often at low resource density and small cluster size, whereas foray loops were observed more often at high resource density and the proportion of foray loops generally increased with decreasing number of clusters (increasing cluster size).

### 3.3 Spatially structured population properties of the system

**FIGURE 5:**
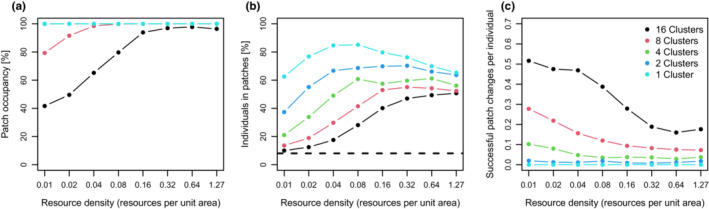
(a) Averaged percentage of patch occupancy, (b) averaged percentage of individuals in clusters, and (c) average number of successful patch changes per individual (all values were calculated during the last 2000 time steps) plotted over resource density. The dashed line in (b) shows the expected percentage of individuals in clusters if they were randomly distributed.

Generally, we could observe attributes of a spatially structured population in our system as described above, i.e., spatially clustered distribution of individuals, different movement pattern inside and outside patches, and emigration rate depending on patch quality and size. We found that in most scenarios, patch occupancy was almost 100%. The mean patch occupancy was lower than 85% only in five scenarios, i.e., with eight clusters and resource density *u* = 0.01, and in scenarios with 16 clusters and *u* ≤ 0.08 (Figure 5a), scenarios where the distribution of individuals across the whole landscape was still nearly random, i.e., the proportion of individuals in clusters nearly matched the ≈8% that are expected under a random distribution of individuals (Figure 5b). With increasing resource density and cluster size, individuals increasingly concentrated within resource clusters (“habitat”). For example, if 50% of individuals reside inside resource clusters that cover just 8% of the total area, the “population density” inside cluster is already 11.5 times larger than in the surrounding matrix. In passing, we note that these results completely deviate from those predicted by the diffusion approximation outlined by Turchin (1991); see also Patlak (1953a, 1953b); for more details on underlying reasons, see discussion. However, for the scenarios with few clusters, the response to resource density was unimodal due to the increasing emigration probability mentioned before. The highest number of successful patch changes per individual was observed in scenarios with many clusters and low resource density, and this value decreased with lower number of clusters and higher resource density (Figure 5c) and was almost or equal to zero at *k* < 2.

**FIGURE 6:**
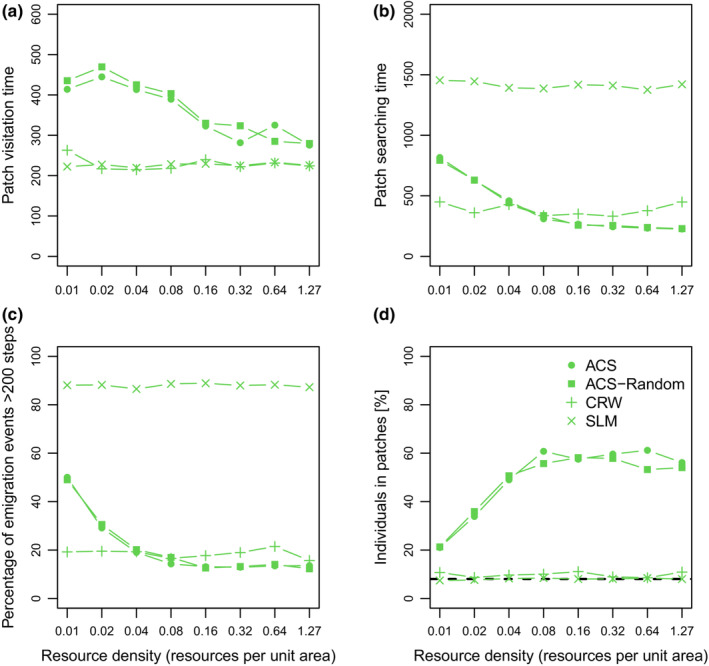
Comparison between area‐concentrated search (“ACS” stands for scenarios with starting points within clusters and “ACS‐Random” for scenarios with random starting points), correlated random walk (CRW), and straight‐line movement (SLM) in four‐cluster scenarios. (a) Averaged duration of visits to a resource cluster (patch visitation time) and (b) averaged duration of patch searches (patch searching time) during the last 2000 time steps plotted over resource density. (c) The percentage of emigration events that were longer than 200 steps (permanent emigration) plotted over resource density; (d) averaged percentage of individuals residing in clusters. Note that the two ACS scenarios with individuals released within clusters (standard) or at random coordinates lead to nearly identical results.

Our results are qualitatively robust against changes in parameters *α* and *h* over a wide range of parameter values. However, in the additional simulations with either straight movement (SLM; *h* = 0) or a simple CRW (*h* = 10.000) in four‐cluster scenario, we found that the spatial patterns described above completely disappeared as did any dependence on resource density that emerges for the ACS (Figure 6). Individuals with ACS and ACS‐Random stayed longer in patches (Figure 6a) and immigrated after shorter time than individuals following SLM (Figure 6b). Looping occurred quite frequently with ACS at higher resource densities resulting in briefer periods between emigration and immigration (Figure 6c); looping occurred rarely with SLM and frequently for CRW but was not dependent on resource density for either of the latter two. In the ACS and ACS‐Random scenarios—which both generate nearly identical results—more individuals resided within resource clusters, whereas in scenarios with CRW and SLM, the proportion of individuals within patches was not above the random expectation (~8%) at any resource density (Figure 6d).

## 4 DISCUSSION

In our simulations, we use a simple movement model (ACS) on the one hand and landscapes with spatially concentrated resource distribution on the other to simulate behavior of foraging individuals; the simulated populations show attributes of a spatially structured population as emergent properties. As such, the emergence of spatial structure cannot be a very surprising outcome as it is already an intrinsic property of the ACS that individuals tend to preferentially stay in areas of high resource concentration (e.g., Dorfman et al., 2022). At the population level, this would make us expect that animals tend to concentrate in areas where critical resources are aggregated; in our simulations, population densities were up to approx. 65 times larger inside clusters than outside (Figure 5b). This observation here is similar to the work by Turchin (1991) who in fact provided a one‐dimensional solution for the problem. Nonetheless, we see a value in our simulations in making clear that neither the perception of a patch‐matrix dichotomy nor spatial memory or any complex decision rules for emigration are needed to generate spatial heterogeneity in the distribution of individuals. Further, the simulations implemented here also generate more specific patterns that are expected to emerge in spatially structured population systems, i.e., that individuals are more likely to emigrate from small vs. large resource clusters (viz. patches) and with greater probability from poor quality (low resource density) than from high‐quality clusters (but see below). The control simulations with SLM or CRW indeed show that the spatial patterns reported only emerge with the ACS but not with movement rules that show no dependence on the individuals' experience.

### 4.1 Foraging behavior and foraging success

As expected, a reduction of the number of clusters (larger cluster at the same time) and/or an increase in resource density leads to more foraging success of each individual and also affects movement pattern of individuals. In the scenarios with high resource density or larger cluster, individuals tend to stay long within a patch and perform more area‐concentrated search than straight‐line movement. Such effects of resource density and resource spatial arrangement on movement strategies and foraging success were also observed in previous studies (Bartoń & Hovestadt, 2013; Benhamou, 1992; Kareiva & Odell, 1987; Nolting et al., 2015; Scharf et al., 2009). Note that in our scenarios, the tendency to remain in a resource aggregation is only driven by the attributes of the ACS but does not require that individuals respond to or even recognize (suitable) habitat per se as is the underlying assumption in Turchin (1991). It also does not require that individuals apply different rules of movement to habitat and matrix or that individuals ever take a decision to emigrate from a habitat patch. Saying so, we do not want to exclude and even suggest that animals typically forage with more sophistication than we assume in our model, e.g., that they utilize environmental cues, e.g., habitat suitability, that indicate that finding resources would be more likely in a certain region or base movement decisions on experience and spatial memory (as examples in Avgar et al., 2013; Fronhofer et al., 2013); any of such movement rules will create ACS like movement trajectories leading to a concentration of individuals in regions of resource concentration.

Interestingly, the greatest foraging success occurred in scenarios with a single resource cluster and highest resource density, but individuals did not stay longest within patches in this scenario: contrary to expectation, the longest residence times were observed in scenarios with moderate resource density, but the peak in this relationship also depends on the number of clusters. An underlying reason is that individuals tended to stay nearer to patch edges if resource density was very high and did not move as far into a patch (approaching the patch center) compared to individuals in scenarios with moderate resource density; the underlying reason is that switching to searching behavior typically occurred already near the patch border if resource density was high. Therefore, they tended to leave patches more often than in the other scenarios. Particularly with high resource concentration, many emigrations resembled foray loops, however, where individuals return to the same patch (Conradt et al., 2003; McIntire et al., 2013). On the other hand, with very low resource density, individuals often moved through resource clusters without encountering (perceiving) resources at all and consequently maintaining a very directed walk, leaving the patch quickly again. Emigration events as well as foray loops might become rarer if individuals were to apply more sophisticated movement rules than those implemented here, e.g., when perceiving habitat per se, using memorized knowledge about patch location (Avgar et al., 2013; Fagan et al., 2013; Fryxell et al., 2008; Van Moorter et al., 2009), knowledge about patch quality (Olsson & Brown, 2010), improved perception range (Avgar et al., 2013; Johnston & Painter, 2019), or applying smarter Bayesian movement decision rules (Fronhofer et al., 2013). Indeed, in preliminary simulations, we found that a simple ACS with a delayed switching in movement randomness after encountering a resource item resulted in deeper penetration into resource clusters and longer patch residence times. Adding any of such behavioral components might lead to edge “avoidance” and a more “organized” and efficient resource utilization from clusters and should lead to a decrease in emigrations and foray loops in scenarios with high resource density.

### 4.2 Spatially structured population properties of the system

We show that our system with simple area‐concentrated search develops properties of a spatially structured population over a wide parameter range. We find interesting interaction effects between number of resource clusters and resource density on the one hand and emerging population density inside and outside aggregations on the other. Our findings thus completely deviate from those predicted by Turchin (1991) who based predictions on a one‐dimensional model where individuals modulate directionality of movement based on the perception of habitat per se, i.e., whenever they enter an area designated as habitat. In our simulations, however, they only change movement once they encountered a resource item. In fact, with the constant values for step length and duration as assumed in our simulations, Turchin's analytical equations predict an even density of individuals inside and outside habitat—a prediction we could validate by implementing simulation rules that exactly match those assumed by Turchin. To some degree, the difference in our findings and those predicted by Turchin may be a consequence of us implementing circular resource cluster, whereas Turchin assumed a one‐dimensional transition between habitat and non‐habitat (i.e., habitat stripes) but we think that the far more important reason for the difference between our findings and Turchin's predictions is the difference in the movement rules implemented—changing movement directionality (to lower) when encountering habitat in Turchin's model but only changing directionality the moment individuals detect a resource item in our model. Turchin's model thus assumes a principal “awareness” of habitat per se, whereas we do not make such an assumption; consequently, a dependence of residence time on resource density cannot emerge in Turchin's model, whereas in our model, individuals will often enough never switch to the search mode when entering a low‐density patch.

The number of successful patch changes (emigration from one and immigration into another resource patch) was indeed quite low (Figure 5c), but these values can only be interpreted in relation to the total period covered by our scenarios. For example, if we assume that a single time step in this simulation is 5 min, the 2000 time steps analyzed cover a period of approximately 7 days. Further assuming that animals are active only 12 h a day (e.g., because they are nocturnal), the period covered would correspond to *c*. 2 weeks, a value that is reasonable for the expected life span of many adult insects. Based on these assumptions, we thus find that in many of our simulations, only a small fraction of individuals (mostly <20%) successfully “dispersed” from one habitat cluster to another during their lifetime.

In this study, we varied patch structure (many small clusters to single large cluster and low to high resource density), but within a scenario, all patches had identical properties. Creating landscapes with resource clusters of variable attributes might enable us to investigate the emergence of spatial structure in populations in other landscape settings, e.g., settings that show attributes of a mainland–island system (Harrison, 1991) or a system with varying patch quality (resource density) like in source–sink systems (Pulliam, 1988). It must also be mentioned that we did not demonstrate the effect of the two parameters α and *h* (apart from the extreme values for *h* that change movement fundamentally), but previous studies showed that our choice of parameter values is adequate to result in good foraging success in a broad spectrum of parameters for the spatial distribution of resources (cf. Bartoń & Hovestadt, 2013 for more details). Generally, a decrease of the half‐saturation constant *h* should lead to an increase in emigration rates and a reduction of patch residence times as we increasingly approach straight‐line movement.

This is also supported when contrasting the ACS movement results with those of the CRW and the SLM. At very low resource density, ACS behavior becomes more or less similar to the SLM as individuals rarely switch into the search mode. On the other hand, at very high resource density, individuals will usually change to the CRW behavior more or less immediately after entering a patch. And this will also be true in many cases when leaving a patch again, explaining the similarity between the emigration “attributes” between ACS and CRW results seen in Figure 6b,c at high resource densities.

In this model, we assumed no birth and death events in the population because we simulated only short ecological time interval and we avoided complexity caused by birth and death process, such as population dynamics. By excluding natality and mortality, we also did not include factors that might affect the spatial structure, such as dispersal mortality, starvation, or environmental stochasticity (Chaianunporn & Hovestadt, 2012, 2019; Fronhofer et al., 2012). Including these factors, emigration rates and spatial population structure in this system would presumably change. In addition, more realistic models should in fact also account for a proper resource dynamic, e.g., by either simulating abiotic resources with a constant supply rate (patch specific) or by implementing it as a prey population with its own population dynamics.

## 5 CONCLUSIONS

In this study, we implement a model of organism with area‐concentrated search as a foraging movement rule moving in a continuous landscape with aggregated resource distribution. Although we do not include population dynamics (birth and death) into the system, the simulated collective of individuals expresses properties of spatially structured populations as emergent properties. Models like this can be used to improve our understanding of the mechanisms underlying the emergence of population spatial structure but could also be applied—given we know the rules of movement—to foresee the effects of landscape changes viz. changes in resource distribution on (endangered) populations. Furthermore, the model could be extended by adding components that affect population dynamics, e.g., dispersal mortality, environmental stochasticity, heterogeneous patch quality, or varying natality and mortality, to gain more understanding about population change in heterogeneous landscape. Mechanistic models like ours may help to close the gap between individual orientated movement ecology and population oriented spatial ecology theory.

## AUTHOR CONTRIBUTIONS

**Thomas Hovestadt:** Conceptualization (equal); funding acquisition (equal); methodology (equal); project administration (equal); supervision (lead); validation (equal); writing – review and editing (lead). **Thotsapol Chaianunporn:** Conceptualization (equal); data curation (lead); formal analysis (equal); funding acquisition (equal); investigation (lead); methodology (equal); project administration (equal); resources (equal); software (lead); validation (equal); visualization (lead); writing – original draft (lead).

### OPEN RESEARCH BADGES

This article has earned Open Data and Open Materials badges. Data and materials are available at https://doi.org/10.5061/dryad.n8pk0p2xr and https://doi.org/10.5281/zenodo.6498038.

## Data Availability

The data and the code of the simulation model that support the findings of this study are openly available in “Dryad” at https://doi.org/10.5061/dryad.n8pk0p2xr.
